# Recommendations of the Brazilian Society of Nephrology for regulating access to outpatient dialysis in the Brazilian Unified Health System

**DOI:** 10.1590/2175-8239-JBN-2025-0261en

**Published:** 2026-02-06

**Authors:** Farid Samaan, Fernanda Salomão Gorayeb-Polacchini, Alexandre Minetto Brabo, Paulo Henrique Fraxino, Fábio Humberto Ferraz, Ana Lydia Lédo de Castro Ribeiro Cabeça, René Scalet dos Santos, Patrícia Ferreira Abreu, José A. Moura-Neto

**Affiliations:** 1 Sociedade Brasileira de Nefrologia, São Paulo, SP, Brazil.; 2 Secretaria de Estado da Saúde de São Paulo, São Paulo, SP, Brazil.; 3 Instituto Dante Pazzanese de Cardiologia, São Paulo, SP, Brazil.; 4 Hospital de Base de São José do Rio Preto, São José do Rio Preto, SP, Brazil.; 5 Hospital de Base de Bauru, Bauru, SP, Brazil.; 6 Conselho Regional de Medicina, Curitiba, PR, Brazil.; 7 Universidade do Distrito Federal, Brasília, DF, Brazil.; 8 Hospital de Clínicas Gaspar Vianna, Belém, PA, Brazil.; 9 Secretaria Municipal de Saúde de Curitiba, Curitiba, PR, Brazil.; 10 Universidade Federal de São Paulo, São Paulo, SP, Brazil.; 11 Escola Bahiana de Medicina e Saúde, Salvador, BA, Brazil.

**Keywords:** Unified Health System, Health Care Coordination and Monitoring, Health Services Accessibility, Ambulatory Care, Renal Replacement Therapy, Renal Insufficiency, Chronic, Consensus

## Abstract

The increase in chronic kidney disease prevalence and its risk factors have pressured universal health systems to expand the supply of kidney replacement therapy (KRT - hemodialysis, peritoneal dialysis and kidney transplantation). Particularly in low- and middle-income countries and those undergoing a fast epidemiological and demographic transition, the access to nephrology consultations and multidisciplinary care is limited, and the majority of patients start KRT in an unplanned manner or during emergency hospitalization. Even patients with adequate pre-dialysis care and elective requests for KRT are at risk of clinical decompensation and requiring hospitalization to start emergency dialysis; this risk increases the longer the delay in starting KRT. In both cases, the patient’s access to an outpatient dialysis unit must be timely and the transition of care safe. There are Brazilian and international guidelines for patients who are prevalent on dialysis. However, there are no clear recommendations for regulating access to the start of outpatient KRT, which often leads to divergent opinions among healthcare professionals and contributes to the inefficiency of the regulatory process. This document aims to: (1) list the main challenges in the daily practice of the regulatory professionals in the Brazilian Unified Health System; (2) present recommendations from the Brazilian Society of Nephrology based on scientific evidence and available legislation.

## Introduction

The increasing prevalence of chronic kidney disease (CKD) and its associated risk factors has put pressure on countries with universal healthcare systems to expand the availability of kidney replacement therapy (KRT: hemodialysis [HD], peritoneal dialysis [PD], and kidney transplantation)^1^. Among these countries, Brazil stands out for having the fourth largest population undergoing chronic dialysis (HD or PD) worldwide and being one of the ten nations with the highest incidence of individuals affected by this condition^2^.

Tackling CKD is particularly challenging for countries undergoing rapid epidemiological and demographic transition^3,4^. In Brazil, over the past three decades, the prevalence of adults with hypertension (HTN) has increased from 21% to 28%, diabetes mellitus (DM) from 5% to 9%, and obesity from 11% to 23%^5^. During the same period, the proportion of individuals aged 60 years or older rose from 9% to 13% of the population^6^.

It is known that the transition from conservative treatment of CKD to the initiation of chronic dialysis is particularly critical due to physical, psychological, and social changes experienced by patients, as well as the consequent increased risk of hospitalization, death, and use of healthcare resources^7^. In Brazil and in several other countries, shortcomings in the implementation of the CKD standard of care^8,9^ mean that the initiation of KRT is mostly unplanned, occurring during urgent/emergency hospitalization^10,11^. Even patients undergoing predialysis care and elective requests for KRT may experience clinical decompensation and require hospitalization, especially if the time to dialysis initiation is prolonged. In both cases, it is essential that the patient’s access to an outpatient dialysis unit occurs as promptly and safely as possible.

There are guidelines that address best practices for the management of prevalent dialysis patients^12,13^. Nevertheless, the administrative processes governing patient access to outpatient dialysis are not standardized and are a source of disagreement among the professionals involved, namely public health administrators, attending physicians, regulatory physicians, and KRT service providers. Therefore, the objectives of this document are as follows: (1) to outline the main challenges encountered in the daily practice of professionals who regulate outpatient dialysis slots within the Brazilian Unified Health System (SUS, in Portuguese); and (2) to present recommendations from the Brazilian Society of Nephrology (SBN) that are grounded in available evidence and legislation. The initiative is a response to the growing need for organization and transparency in regulatory workflows, in view of the high demand from the population and the logistical complexity involved in nephrology care in Brazil.

## Methods

### Stages of Consensus Development

#### Identification of Problems

Identification of situations with potential for divergent opinions among professionals involved in the regulatory process, resulting in impaired timely and safe patient access to outpatient dialysis units. At this stage, members of the SBN (Executive Board and Dialysis Department) were consulted, including at least one representative from each region of Brazil.

#### Development of Recommendations

Whenever possible, recommendations were developed based on scientific evidence and Brazilian legislation. Some situations identified as potential sources of divergent opinions had a technical nature, which allowed the recommendation to be supported by specialized literature and current standards; others, however, were essentially managerial in nature, requiring the development of suggestions based on the authors’ experience.

#### Participation of the SBN Regional Units

All 19 SBN regional units that were active in June 2025 received the initial document for free comments (Alagoas, Bahia, Ceará, Federal District, Espírito Santo, Goiás, Maranhão, Minas Gerais, Mato Grosso, Pará, Paraíba, Paraná, Pernambuco, Piauí, Rio Grande do Norte, Rio Grande do Sul, Rio de Janeiro, Santa Catarina, and São Paulo). The purpose of this phase was to ensure that the recommendations guide represented, as accurately as possible, the diverse realities of access to outpatient KRT across Brazil.

#### Public Consultation

The first version of the recommendations was made available for public consultation and published on the Brazilian Society of Nephrology’s website and social media pages. Respondents were invited to access an electronic form, in which they indicated their position in categorical terms (“agree,” “partially agree,” or “disagree”) and were provided with an open-text field to record their contributions.

### Definitions Used

#### Outpatient KRT

In this consensus, the terms “outpatient KRT” and “outpatient dialysis” were used interchangeably to refer to hemodialysis and/or peritoneal dialysis procedures performed in healthcare facilities operating under the Ministry of Health’s licenses 1504 and 1505^13^. The term “outpatient” was placed before “KRT” or “dialysis” because procedures performed in the SUS are classified into two treatment modalities—outpatient and inpatient—and the scope of this document is the regulation of access to outpatient procedures. Furthermore, it was deemed appropriate to use the expressions “outpatient dialysis” and “outpatient KRT” as opposed to the terms “inpatient dialysis,” “inpatient procedures,” and “inpatient artificial renal support,” used in the Guidelines for Hospital Nephrology Assistance from the Brazilian Society of Nephrology^14^.

#### Dialysis Unit

Healthcare facility where hemodialysis and/or peritoneal dialysis procedures are performed and whose operation is regulated under the Ministry of Health’s licenses 1504 and 1505^13^.

#### Professionals Involved in the Regulation Process

- Requester: physician requesting an outpatient KRT slot (HD or PD).
- Regulator: physician working in the SUS management system who evaluates dialysis requests, verifies their technical relevance and the presence of mandatory documents, and forwards the case to be evaluated at a dialysis unit.
- Performer: physician at the dialysis unit who evaluates the patient and their tests and determines whether the patient will be admitted to the service.

#### Types of Outpatient KRT Requests

- Initial: request for patients who are not enrolled in a chronic dialysis program. These patients may or may not be hospitalized.
- Modality change: request for change between the modalities offered within the SUS: HD, APD (automated peritoneal dialysis), CAPD (continuous ambulatory peritoneal dialysis), and IPD (intermittent peritoneal dialysis).
- Transfer: request for patients who wish to change their treatment to another dialysis unit.
- Transit: request to undergo hemodialysis in another municipality or state for a period of up to 30 days.

## Results

Fourteen scenarios with potential for divergent opinions among professionals involved in the regulatory processes for outpatient KRT access were identified. The public consultation on the initial document received contributions from 20 individuals, of whom nine (45%) agreed with the recommendations; nine (45%) partially agreed and offered suggestions; and two (10%) disagreed without providing specific comments. One of the received contributions suggested an additional scenario with potential regulatory uncertainty to the 14 initially identified. After analysis and review by the authors, 58% (14/24) of the suggestions were incorporated, and 15 recommendations were developed.

### Regulation of Cases with Suspected or Confirmed Hepatitis B or C

**Recommendation 1.** Patients with elevated ALT/AST levels of undetermined cause and non-reactive HBsAg and/or anti-HCV require HBV-DNA and/or HCV-RNA testing prior to admission to a dialysis unit, regardless of the type of request (initial, modality change, transfer, or transit). Similarly, individuals with suspected occult hepatitis B (reactive total or IgM anti-HBc, with non-reactive anti-HBs), even in the absence of elevated transaminases, should undergo viral load testing. In such cases, the obligation to perform confirmatory tests falls on the physician requesting the slot.

**Comment**. Patients with advanced CKD usually have low serum transaminase levels (alanine aminotransferase [ALT] and aspartate aminotransferase [AST])^15^. In this population, any elevation in ALT or AST above reference values or ≥ 50% above baseline requires investigation^16^. The main causes of elevated transaminases include drug-induced toxicity, muscle injury, and infections, among others^15^.

Among infections in HD patients, hepatitis B and C stand out due to occupational risk and the possibility of cross-transmission within dialysis units, their potential severity, and the availability of specific treatment^17,18^. The “immunological window” is commonly defined as the interval between infection by a given biological agent and its detection during routine testing. In hepatitis B, this interval is 30-60 days until the hepatitis B surface antigen (HBsAg) becomes reactive. In hepatitis C, the period between infection and detection of antibodies against the C virus (anti-HCV) ranges from 50 to 70 days.

In cases where immediate diagnosis is imperative, such as upon admission to dialysis units, tests capable of detecting an ongoing infection in real time are indicated, i.e., viral load (quantitative or qualitative), also referred to as polymerase chain reaction (PCR), which identifies particles of the B virus (DNA) or C virus (RNA) in the blood or other bodily fluids^16^.

To regulate access to KRT for individuals who are anti-HCV-reactive and/or HBsAg-reactive, it is recommended that, whenever possible, the requester perform molecular testing prior to the patient’s admission to the dialysis unit^17,18^. It is also recommended that these tests (HCV-RNA and HBV-DNA viral load) be available in dialysis units, with coverage by the SUS, for both diagnostic and followup purposes.

It should be noted that, in the case of hepatitis C, the diagnosis of active disease could only be established through the detection of viral load, unlike hepatitis B, in which the presence of reactive HBsAg is sufficient to confirm an ongoing infection^16,17^. While dialysis patients with reactive HCV-RNA have high cure rates once appropriate treatment is instituted, those with confirmed hepatitis B may require medications with a higher risk of adverse events and a lower rate of sustained response^17,18^.

### Hospitalized Patient Medically Fit for Discharge, Asymptomatic, and with a Positive Surveillance Culture for Multidrug-Resistant Bacteria (MDR)

**Recommendation 2**. Admission to dialysis units should not be delayed pending negative results from positive surveillance cultures for MDR in hospitalized, asymptomatic patients who are medically fit for discharge. In these healthcare facilities, patients should receive standard contact precautions, and there is no requirement to perform HD in an isolation room.

**Comment**. According to recommendations from the Brazilian Health Regulatory Agency (ANVISA), all dialysis services must have protocols for infection prevention and control, as well as for monitoring and reporting adverse events (including healthcare-associated infections)^19^. When caring for patients colonized/infected with MDR organisms, the use of isolation rooms, separate hemodialysis equipment, and dedicated staff is not mandatory. Preventive measures must be strictly followed: proper hand hygiene, cleaning and disinfection of surfaces, use of gloves and gowns for all contact with the patient, and use of goggles and masks to protect the mucous membranes of the eyes, mouth, and nose of healthcare professionals, among other measures^19,20,21^.

### Patient Admitted to Private Hospitals, Medically Fit for Discharge, with a Request for an Outpatient KRT Slot in the SUS

**Recommendation 3**. Patients should not be transferred to a public hospital for the sole purpose of formalizing a request for outpatient KRT, except when expressly desired by the individual or their legal guardian. In private hospitals, requests for outpatient KRT slots in the SUS must be forwarded to the Municipal Health Secretariat (SMS) of the patient’s destination. The SMS is responsible for informing the requesting hospital of the required documents, clinical data, and mandatory tests, in accordance with local regulations.

**Comment**. Patients admitted to private hospitals could rely exclusively on the SUS (as in the case of kidney transplant patients treated in private hospitals that provide services to the SUS, for example), have private health insurance, or choose to cover hospitalization costs out of pocket. For hospitalized patients who have private health insurance, the private hospital should submit a request for a KRT slot in the SUS to the Municipal Health Secretariat (SMS) only after all possibilities for arranging KRT care between the health insurance plan and a dialysis unit have been exhausted. Regardless of these scenarios, access to the SUS is a universal right in Brazil, guaranteed to every citizen throughout the national territory. The recommendation establishes an access flow to outpatient KRT in private healthcare facilities, especially when these services do not have access to the SUS regulatory processes. The rationale for recommending that the municipal authority be the recipient of outpatient KRT requests is supported by the principle of SUS decentralization and by the current ministerial ordinance^13,22^. It is the hospital’s responsibility to send all clinical information and documentation to the SMS in accordance with the SUS standards of the municipality where the KRT slot is being requested^13^. The SMS of the patient’s destination municipality must receive the complete documentation and include the case in the local regulatory processes^13^. Clear and continuous communication between the private hospital and the public regulatory authority is essential.

### Person Receiving Outpatient KRT Through Private Health Insurance Whose Supplementary Coverage is Interrupted

**Recommendation 4**. It is not recommended to refer any patient undergoing KRT to hospital admission solely due to the voluntary or involuntary cancellation of their private health insurance plan. Once all possibilities for contracting KRT care between the private health plan and the dialysis unit have been exhausted, the facility should submit a request for a KRT slot in the SUS to the Municipal Health Secretariat (SMS, in Portuguese) as promptly as possible.

**Comment**. There are different ways of contracting private health plans (individual, family, or employer-sponsored) and various reasons for terminating these contracts (non-payment, plan change, beneficiary exclusion, job termination, etc.). In each situation, the period during which healthcare coverage must be maintained by the respective plan may vary according to the rules established in the contracts and guaranteed by the National Supplementary Health Agency. In general, this period ranges from 30 to 60 days^23,24,25^. Thus, to minimize revenue losses, it is recommended that dialysis units keep updated information on the terms of contracts signed between the patients and the private health insurance plans.

### Patient Requesting an Outpatient KRT Slot un the SUS Coming from Another State or Country

**Recommendation 5**. The request for a KRT slot within the SUS must be submitted by the healthcare service of the patient’s state or country of origin directly to the SMS of the requested destination. The SMS is responsible for informing the requester of the local regulations, including the complete list of mandatory documents, clinical information, and exams.

**Comment**. The SUS principle of universality ensures that access to healthcare services is a right of all Brazilian citizens and all individuals within the national territory. This includes Brazilians residing in other states, as well as foreigners, migrants, immigrants, refugees, and asylum seekers^22,26^. The recommendation to forward elective outpatient KRT requests directly to the destination SMS aims to streamline the workflow, enabling public authorities to include the case in the regulatory queue in the same manner they would for their own residents^13,22^. In emergency situations, the Ministry of Health recognizes the right to healthcare even for individuals who do not possess a Brazilian tax identification number (CPF) or a national identity card (RG)^22^.

### Should Certain Types of Outpatient KRT Requests be Prioritized Over Others?

**Recommendation 6**. It is recommended that the following descending order of priority be followed: (1) initial request for hospitalized patients; (2) request for modality change; (3) initial request for home-based patients; and (4) request for transfer or transit. For patients within the same situation among those listed above, it is suggested that priority criteria be ordered from the oldest to the most recent.

**Comment**. The ordering of access to healthcare services using criteria other than the chronological order of requests is a standard practice within the SUS and is part of what is commonly referred to as queue management or queue qualification. Examples of the application of clinical priority criteria in healthcare service queues may be observed in outpatient care^27,28,29,30^, inpatient care^31^, and in the context of KRT^32^. The beneficial consequence of these management processes is that different average waiting periods are established according to the patient’s current condition and the presumed risk of clinical deterioration.

Patients who are hospitalized merely to wait for an outpatient KRT slot are at high risk of malnutrition, hospital-acquired infections, prolonged hospitalization, and death^33,34,35,36,37^. Moreover, the idle occupation of a hospital bed hinders access for other patients and increases the operating costs of the healthcare system (priority 1).

Requests for outpatient KRT modality changes — from hemodialysis to peritoneal dialysis — are often due to vascular access failure, recurrent bloodstream infections, and hemodynamic instability^35^; switching from peritoneal dialysis to hemodialysis may occur due to peritonitis or loss of peritoneal membrane exchange efficiency^38,39,40^. In both cases, there is significant vulnerability and an imminent risk of clinical deterioration (priority 2)^41,42^.

It is acknowledged that individuals with an elective request for outpatient KRT who are at home remain at risk of sudden worsening of kidney function and subsequent hospitalization for urgent dialysis. This risk increases as the waiting period for KRT initiation lengthens. However, given the nature of the request, the clinical condition of these individuals is less severe than the conditions described in priorities 1 and 2. Before CKD in individuals with an elective KRT request worsens, these patients may still consult their original outpatient service for adjustments to their therapeutic plan. Accordingly, they should remain under follow-up at the service in question for the purpose of continuous therapy adjustments until a KRT slot becomes available (priority 3).

Timely processing of outpatient KRT requests for transit or transfer is undoubtedly essential for several reasons; nevertheless, the clinical condition of the applicants is more stable than in initial and modality change requests. It is therefore suggested that transit or transfer requests be classified as priority 4.

### Should the Performer Repeat the Viral Serologies Sent by the Requester (Hepatitis B and C, and Hiv) Before Admission to the Dialysis Unit?

**Recommendation 7**. It is not recommended that the performer repeat viral serology tests (hepatitis B and C, and HIV) sent by the requester routinely or without valid justification.

**Comment**. The patient’s primary healthcare services are directly responsible for performing and validating the tests conducted. The public agencies that oversee compliance with operating standards and quality control for clinical analysis laboratories (both public and private) are the state and municipal health surveillance centers^43^. The requirement to repeat valid serological tests impairs the regulatory flow of slots, delays the initiation of KRT, may negatively impact patient health, and constitutes an inappropriate use of resources, in addition to representing increased costs for the healthcare system. The list of mandatory exams for admission to a dialysis unit and the recommended validity periods are described in recommendation 8 of this consensus.

### In Outpatient KRT Requests, Which Exams Must be Sent by the Requesting Physician to the Regulating Physician?

**Recommendation 8**. It is the requester’s responsibility to send the agreed-upon test results and to comply with a maximum period between the test results and the date of evaluation by the performer. For initial requests involving hospitalized patients, the following are recommended: complete blood count, urea, creatinine, sodium, potassium, blood glucose, calcium (total or ionic), and ALT (valid for 15 days); anti-HCV, anti-HBc-total, HBsAg, anti-HBs, anti-HIV, and renal ultrasound (90 days). For initial requests related to patients who are at home, the following are recommended: complete blood count, urea, creatinine, sodium, potassium, blood glucose, calcium (total or ionic), and ALT (60 days); anti-HCV, anti-HBc-total, HBsAg, anti-HBs, anti-HIV (180 days), and kidney ultrasound (360 days). For transit or transfer requests, it is recommended to send the most recent tests performed at the dialysis unit of origin. These should be valid for a maximum period equivalent to the frequency provided for in the current Brazilian guidelines, that is: hemoglobin, hematocrit, creatinine, sodium, potassium, calcium (ionic or total), phosphorus, ALT, urea (pre and post), and blood glucose (for people with diabetes mellitus) - 30 days; complete blood count, transferrin saturation, ferritin, alkaline phosphatase, parathyroid hormone, and glycated hemoglobin (if diabetes) - 90 days; anti-HCV, anti-HBc-total, HBsAg, anti-HBs - 180 days, and anti-HIV - 360 days^44^.

**Comment**. In the case of initial requests for outpatient KRT, the tests recommended in the regulatory process must ensure that the performer can provide a dialysis prescription that is safe for the newly admitted patient and for other patients in the receiving dialysis unit. Mandatory tests should, at a minimum, allow confirmation of the diagnosis of CKD (urea, creatinine, kidney ultrasound)^12,13,44^, prevention of arterial hypotension (hemoglobin/hematocrit)^45^, bleeding (platelet count)^46^, and electrolyte disorders (sodium, potassium, calcium)^47^, in addition to enabling the reduction of occupational biological risk and cross-transmission of hepatitis and HIV (viral serology)^16,17,18,48,49^.

### Which Documents Must be Provided to the Regulatory Physician by the Physician Requesting Outpatient KRT?

**Recommendation 9**. It is the responsibility of the requester to send a medical report and the results of the mandatory tests, in accordance with recommendation 8. The medical report must be comprehensive, progressive, and up to date, including clinical history, current medications, physical examination findings, and, in cases where the patient has already initiated KRT, a history of dialysis accesses (catheters [venous and peritoneal] and arteriovenous fistula [AVF]), as well as the medical prescription for the procedure (HD or PD).

**Comment**. It is the requester’s responsibility to provide all clinical information, laboratory tests, and mandatory documents agreed upon in the regulatory workflow. Incomplete submission of this information delays acceptance of the patient by the executing unit, postpones the timely initiation of dialysis treatment, and hinders the formulation of a safe treatment prescription at the new dialysis unit^45,46,47^.

### Which Exams and Documents May be Waived from the Regulatory Process for Access to Outpatient KRT?

**Recommendation 10**. For the purposes of admission to a dialysis unit, the requester should not be required to provide the results of exams such as phosphorus, parathyroid hormone, total cholesterol and fractions, triglycerides, iron levels, ferritin, transferrin saturation, electrocardiogram, echocardiogram, chest X-ray, syphilis serology, among others. Similarly, admission should not be conditional upon the presentation of the patient’s vaccination card or any tests other than those indicated in recommendations 8 and 9 of this document.

**Comment**. Recommendations 8 and 9 of this consensus state that the minimum number of documents and tests enabling the performer to formulate a safe dialysis prescription should be required. The tests and documents required in the regulatory process for access to KRT are not intended to exhaust the demands of stage 5 CKD patients. These demands should be met after admission to the dialysis unit^12,13,44^.

### In Initial HD Requests, is the Requester’s Responsibility to Perform the Venous Catheter Implantation for HD?

**Recommendation 11**. The requester should not be required to perform venous catheter implantation for HD in hospitalized patients who are clinically stable, medically fit for discharge, not in urgent need of dialysis, and in possession of all mandatory exams and documents. In this case, HD catheter implantation should not be a prerequisite for hospital discharge. Likewise, the dialysis unit should not refuse stage 5 CKD patients who are located at home and do not have a venous catheter or functioning AVF.

**Comment**. The reference for venous catheter implantation and AVF creation must be agreed upon between the public authority and the dialysis units as a prerequisite for the operation of these facilities^13^. These procedures are included in the SUS Table of Procedures, Medications, Orthotics, Prosthetics, and Special Materials (SIGTAP)^50^ and must be provided by the healthcare facility accredited for HD, regardless of whether the patient is located at home or in a hospital^13^.

The purpose of this recommendation is to ensure timely access to a dialysis unit for individuals with stage 5 CKD and should not be interpreted as contradicting the unequivocal concept that HD candidates should ideally undergo AVF creation prior to the imminent need for dialysis initiation^13,44^. Hospitals with the capacity and expertise to perform vascular access procedures could collaborate with the local healthcare system; however, this should not be a condition for patients to be admitted to dialysis units accredited by the SUS.

### In Initial PD Requests for Hospitalized Patients Must Peritoneal Catheter Training and Implantation be Performed Before Hospital Discharge?

**Recommendation 12**. The requester should not be required to perform peritoneal catheter implantation in hospitalized patients who are medically fit for discharge, have not yet initiated KRT, do not need urgent dialysis, and have all mandatory tests and documents. In this case, peritoneal catheter implantation and PD training should not be prerequisites for hospital discharge.

**Comment**. PD training and Tenckhoff catheter implantation are procedures included in the SUS Table of Procedures and must be provided by healthcare services accredited for peritoneal dialysis, regardless of where the patient is located (at home or in a hospital)^13,50^. This recommendation does not apply to patients with any clinical condition that requires hospitalization (hemodynamic instability, severe sepsis, urgent need for dialysis, among others).

### May the Executing Facility Refuse Patients with Comorbidities that Imply Greater Clinical Complexity?

**Recommendation 13**. No clinically stable patient — whether medically fit for hospital discharge or residing at home — who possesses the required tests and documents for regulation may be denied admission solely on the grounds of advanced age or health conditions that fall within the care profile previously agreed upon between health authorities and dialysis units. It is recommended that the care profiles of each unit be clearly defined, respecting structural limitations and ensuring the safety of patient care.

**Comment**. Dialysis services are part of the SUS healthcare system and should not be penalized by being assigned responsibility for the comprehensive treatment of dialysis patients. The entry point for CKD patients, as for all patients within the SUS, is the Primary Healthcare, which is responsible for referring patients to medium and high complexity services and for coordinating comprehensive care^22^. In the event of clinical decompensation during the hemodialysis procedure, the dialysis unit must provide the initial care and ensure patient stabilization^13^. It is the responsibility of the local health authority to ensure immediate access to the urgent and emergency care system, as well as to facilitate coordination between healthcare services, with a view to promoting shared care^13^.

### May the Executing Facility Refuse Patients who are Deprived of their Liberty?

**Recommendation 14**. Admission to a dialysis unit may not be denied or delayed on the grounds that the patient is deprived of liberty.

**Comment**. The National Comprehensive Health Care Policy for People Deprived of Liberty in the Prison System, within the scope of the SUS (PNAISP), aims to guarantee universal, equitable, and continuous access to healthcare actions and services for individuals deprived of liberty^51^. All Brazilian states and the Federal District have formally adhered to the PNAISP and must follow its guidelines, as well as the state and municipal action plans for its implementation.

### What is the Age Limit that Defines Outpatient Dialysis Care for Children?

**Recommendation 15.**Pediatric nephrology care in dialysis units should follow an age limit of up to 12 years complete.

**Comment**. The SUS Management System for the Table of Procedures, Medications, and OPM defines the age range of 0 to 12 years as the target age group for procedures related to pediatric hemodialysis^50^. Accordingly, Ministry of Health Ordinance No. 1675 of 2018, which establishes the criteria for the organization, operation, and financing of care for CKD patients within the SUS, specifies dialysis care, which covers the age range of 0 to 12 years^13^. However, the specific needs of patients with advanced CKD, aged 13 to 18 years old and with low body weight, must be acknowledged. These individuals may require technical adaptations in dialysis units and the assistance of a pediatric nephrologist. It is recommended that this assistance be agreed upon between the contracting public health authority and the dialysis unit.

## Discussion

This consensus yielded 15 recommendations to support the regulation of access to outpatient dialysis within the SUS. During the development and public consultation phases, it was possible to obtain the participation of healthcare professionals from all five regions of Brazil.

There is an urgent need to standardize the regulations governing access to outpatient dialysis. Despite the majority of dialysis units operating under the Brazilian Unified Health System (SUS) being managed at the municipal level^52^, there is significant intermunicipal - and even interstate - movement of individuals seeking KRT treatment^53^. Among other reasons, this is due to the high percentage of small municipalities^54^, Brazil’s continental dimensions, and inequalities in the provision of healthcare services^55^.

The recommendations presented herein should be interpreted with due consideration for Brazil’s regional diversity. In many locations, the scarcity of dialysis units and the constrained physical and human resources available to requesting services and regulatory bodies will undoubtedly result in greater challenges in implementing the entire content of this document^56^. However, as an illustrative example, despite the expected unavailability of HBV and HCV viral load tests, we did not identify a safe alternative to waiving these exams in patients presenting with unexplained transaminase elevation and negative HBsAg and anti-HCV results, respectively.

In addition, the use of teleregulation should be encouraged as a support tool for regulatory teams, especially in regions with a shortage of nephrologists or logistical difficulties. National and international experiences demonstrate the potential of these tools in expanding access and improving management processes^57^.

It is suggested that, in addition to implementing the recommendations, public health authorities incorporate operational indicators that allow for the evaluation of the efficiency of regulatory processes, such as average waiting time (according to type of request, serological profile, patient age, among other variables), the percentage of requests returned due to incomplete documentation, and, where available, the resolution rate of teleregulation^55^. Besides being efficient, it is recommended that access to outpatient dialysis be transparent: the healthcare service requesting the KRT slot must be informed whether or not the request has been fully received by the regulatory center; the patient or their legal representative could request information regarding their position on the waiting list for a KRT slot; both requesters and patients should have the right to know the average waiting time for such placement in a given municipality or health microregion.

Globally, the manner in which individuals initiate KRT reflects, in part, the quality of healthcare delivered at the primary and secondary levels^1,2,4,9,11^. Among others, the following indicators should be publicly available and known to medical societies: the percentage of planned KRT initiation, the rate of HD initiation with functioning AVF, and the percentage of PD use as the first KRT modality. These and other data could be generated from the SUS regulatory systems and are essential for monitoring the standard of care for individuals with CKD^58,59^.

Many of the recommendations contained herein may be applied to contractual agreements between healthcare providers and dialysis units that serve individuals covered by private healthcare insurance plans. It is well established that Brazil has a dual healthcare system^60^, with significant overlap between public and private-sector activities. This reality can be evidenced by the following findings: the regulatory body for both public and private healthcare facilities is the health regulatory agency, an institution belonging to the SUS^13,43^; the right to access the SUS services is universal, regardless of whether or not the individual has a private healthcare insurance^22^; oncology care and KRT (dialysis and kidney transplantation) are the services most used by beneficiaries of private healthcare plans^61,62^.

The regulations governing the mandatory provision of services by health insurance plans are defined by the National Supplementary Health Agency (ANS) and by the specific terms of the contracts entered into with the beneficiary, such as the level of care (outpatient or inpatient, elective or emergency), type of provider facility (owned or accredited), geographical coverage area, among others^24,63^. With the aim of holding private health insurance plans legally and financially accountable, the ANS - an entity that is linked to the Ministry of Health - issues quarterly payment orders for the services provided to their beneficiaries within the SUS system^62^. The amounts not reimbursed by private health insurance plans are converted into outstanding debt in the federal default registry and are subject to legal collection^63^.

In conclusion, it is acknowledged that the entire content of this document does not have the force of law. Nevertheless, it was possible to compile the perspectives of nephrologists from all regions of Brazil and to support most of the recommendations with Brazilian legislation and specialized literature. Therefore, this consensus may support agreements among health authorities, requesters, and performers of outpatient KRT, both within the SUS and in the supplementary health system. Further studies are recommended, preferably conducted in different regions of Brazil, to assess the impact of the proposed recommendations on the efficiency of outpatient KRT access.

## Data Availability

The suggestions received during the public consultation phase, along with the authors’ respective comments, are available upon request to the Brazilian Society of Nephrology, provided reasonable justification is given (secretaria@sbn.org.br).
