# Supplementary material for: Recommendations of the Brazilian Society of Nephrology for regulating access to outpatient dialysis in the Brazilian Unified Health System
Source: J Bras Nefrol. 2026 Feb 6;48(1):e20250261. doi: 10.1590/2175-8239-JBN-2025-0261en (PMC12904151; doi:10.1590/2175-8239-JBN-2025-0261en)
Supplement: Translation

# Translation: Recomendações da Sociedade Brasileira de Nefrologia para regulação de acesso à diálise ambulatorial no Sistema Único de Saúde

**DOI:** 10.1590/2175-8239-JBN-2025-0261pt

**Authors:** Farid Samaan, Fernanda Salomão Gorayeb-Polacchini, Alexandre Minetto Brabo, Paulo Henrique Fraxino, Fábio Humberto Ferraz, Ana Lydia Lédo de Castro Ribeiro Cabeça, René Scalet dos Santos, Patrícia Ferreira Abreu, José A. Moura-Neto

**Affiliations:** 1 Sociedade Brasileira de Nefrologia, São Paulo, SP, Brasil.; 2 Secretaria de Estado da Saúde de São Paulo, São Paulo, SP, Brasil.; 3 Instituto Dante Pazzanese de Cardiologia, São Paulo, SP, Brasil.; 4 Hospital de Base de São José do Rio Preto, São José do Rio Preto, SP, Brasil.; 5 Hospital de Base de Bauru, Bauru, SP, Brasil.; 6 Conselho Regional de Medicina, Curitiba, PR, Brasil.; 7 Universidade do Distrito Federal, Brasília, DF, Brasil.; 8 Hospital de Clínicas Gaspar Vianna, Belém, PA, Brasil.; 9 Secretaria Municipal de Saúde de Curitiba, Curitiba, PR, Brasil.; 10 Universidade Federal de São Paulo, São Paulo, SP, Brasil.; 11 Escola Bahiana de Medicina e Saúde, Salvador, BA, Brasil.

**Keywords:** Sistema Único de Saúde, Regulação e Fiscalização em Saúde, Acessibilidade aos Serviços de Saúde, Assistência Ambulatorial, Terapia de Substituição Renal, Insuficiência Renal Crônica, Consenso

## Abstract

O aumento da prevalência de doença renal crônica e de seus fatores de risco tem pressionado os sistemas universais de saúde a ampliar a oferta de terapia renal substitutiva (TRS — hemodiálise, diálise peritoneal e transplante renal). Sobretudo nos países de renda baixa e média e naqueles em franca transição epidemiológica e demográfica, o acesso a consultas com nefrologista e equipe multiprofissional é limitado, e o início da TRS ocorre majoritariamente de maneira não planejada ou durante internação de urgência/emergência. Mesmo os pacientes em acompanhamento pré-dialítico adequado e com solicitação eletiva de TRS apresentam risco de descompensação clínica e podem requerer hospitalização para iniciar diálise de urgência; esse risco aumenta à medida que se prolonga o tempo de espera para iniciar TRS. Em ambos os casos, o acesso do paciente a uma unidade de diálise ambulatorial deve ser tempestivo e a transição de cuidado, segura. Existem diretrizes brasileiras e internacionais que norteiam o cuidado de pacientes prevalentes em diálise. No entanto, não existem recomendações claras sobre como regular o acesso ao início de TRS ambulatorial, o que frequentemente leva a divergências de opinião entre os profissionais envolvidos e contribui para a ineficiência do processo regulatório. O presente documento tem por objetivos: (1) listar os principais desafios na prática diária dos profissionais de regulação de vagas de diálise ambulatorial no Sistema Único de Saúde; e (2) apresentar recomendações da Sociedade Brasileira de Nefrologia com base em evidências científicas e nas legislações disponíveis.

## Introdução

O aumento da prevalência de doença renal crônica (DRC) e de seus fatores de risco tem pressionado os países com sistemas universais de saúde a aumentar a oferta de terapia renal substitutiva (TRS: hemodiálise [HD], diálise peritoneal [DP] e transplante renal)^1^. Dentre esses países, destaca-se o Brasil, por possuir a quarta maior população em diálise crônica (HD ou DP) do mundo e ser uma das dez nações com a maior incidência de pessoas com essa condição^2^.

O enfrentamento da DRC é, sobretudo, desafiador para países em franca transição epidemiológica e demográfica^3,4^. No caso brasileiro, nas últimas três décadas, a prevalência de adultos com hipertensão arterial (HA) aumentou de 21% para 28%, a de diabetes mellitus (DM), de 5% para 9%, e a de obesidade, de 11% para 23%^5^. No mesmo período, a proporção de indivíduos com 60 anos ou mais cresceu de 9% para 13% da população^6^.

Sabe-se que a transição entre o tratamento conservador da DRC e o início de diálise crônica é especialmente crítica em função de mudanças físicas, psíquicas e sociais dos pacientes, e do consequente aumento do risco de internação, óbito e utilização de recursos em saúde^7^. No Brasil e em diversos países, as falhas de implementação da linha de cuidado da DRC^8,9^ fazem com que o início de TRS se dê majoritariamente de maneira não planejada, durante internação de urgência/emergência^10,11^. Mesmo os pacientes em acompanhamento pré-dialítico com nefrologista e solicitação eletiva de TRS podem apresentar descompensação clínica e requerer hospitalização, especialmente se o tempo até o início da diálise for prolongado. Em ambos os casos, é essencial que o acesso do paciente a uma unidade de diálise ambulatorial aconteça o mais breve possível e de maneira segura.

Existem diretrizes que abordam boas práticas de manejo de pacientes prevalentes em diálise^12,13^. No entanto, os processos gerenciais do acesso de pacientes à diálise ambulatorial não estão padronizados e são fontes de divergências entre os profissionais envolvidos, quais sejam, gestores públicos, médicos assistentes, médicos reguladores e prestadores de serviço de TRS. Portanto, os objetivos deste documento são: (1) listar os principais desafios na prática diária dos profissionais de regulação de vagas de diálise ambulatorial no Sistema Único de Saúde (SUS); e (2) apresentar recomendações da

Sociedade Brasileira de Nefrologia (SBN) com base em evidências e legislações disponíveis. A iniciativa responde à necessidade crescente de organização e transparência nos fluxos regulatórios, frente à elevada demanda da população e à complexidade logística envolvidas na assistência em Nefrologia no Brasil.

## Métodos

### Etapas da Elaboração do Consenso

#### Levantamento de Problemas

Identificação de situações com potencial divergência de opiniões entre os profissionais envolvidos no processo de regulação, com consequente prejuízo ao acesso tempestivo e seguro do paciente a uma unidade de diálise ambulatorial. Nessa etapa, foram consultados membros da SBN (Diretoria Executiva e Departamento de Diálise), incluindo pelo menos um representante de cada região do Brasil.

#### Elaboração de Recomendações

Sempre que possível, as recomendações foram elaboradas com base em evidências científicas e legislações brasileiras. Algumas situações identificadas como potenciais fontes de divergência de opinião apresentaram caráter técnico, o que permitiu que a recomendação fosse amparada por literatura especializada e normas vigentes; outras, entretanto, tinham caráter essencialmente gerencial, demandando a elaboração de sugestões com base na experiência dos autores.

#### Participação das Regionais da SBN

Todas as 19 regionais da SBN ativas no mês de junho de 2025 receberam o documento inicial para manifestações livres (Alagoas, Bahia, Ceará, Distrito Federal, Espírito Santo, Goiás, Maranhão, Minas Gerais, Mato Grosso, Pará, Paraíba, Paraná, Pernambuco, Piauí, Rio Grande do Norte, Rio Grande do Sul, Rio de Janeiro, Santa Catarina e São Paulo). O objetivo dessa etapa foi garantir que o guia de recomendações representasse, ao máximo, as diferentes realidades do acesso à TRS ambulatorial no Brasil.

#### Consulta Pública

A primeira versão das recomendações foi disponibilizada para consulta pública, divulgada nas páginas eletrônicas e redes sociais da Sociedade Brasileira de Nefrologia. Os respondentes foram convidados a acessar um formulário eletrônico, no qual assinalaram seu posicionamento de maneira categorizada (“concordo”, “concordo parcialmente” ou “discordo”) e tiveram espaço para registrar suas contribuições em campo livre.

### Definições Utilizadas

#### TRS Ambulatorial

Neste consenso, adotaram-se os termos “TRS ambulatorial” e “diálise ambulatorial” como sinônimos para designar os procedimentos de hemodiálise e/ou diálise peritoneal realizados em estabelecimentos de saúde cujo funcionamento é regido pelas habilitações 1504 e 1505 do Ministério da Saúde^13^. Optou-se pela palavra “ambulatorial” após “TRS” ou após “diálise” porque os procedimentos realizados no SUS são classificados em duas modalidades de tratamento — ambulatorial e hospitalar — e o escopo deste documento é a regulação de acesso a procedimentos ambulatoriais. Além disso, entendeu-se como apropriada a utilização das expressões “diálise ambulatorial” e “TRS ambulatorial” em oposição aos termos “diálise hospitalar”, “procedimentos hospitalares” e “suporte renal artificial hospitalar”, utilizados no Guia de Assistência Nefrológica Hospitalar da Sociedade Brasileira de Nefrologia^14^.

#### Unidade de Diálise

Estabelecimento de saúde no qual são realizados os procedimentos de hemodiálise e/ou diálise peritoneal e cujo funcionamento é regido pelas habilitações 1504 e 1505 do Ministério da Saúde^13^.

#### Profissionais Envolvidos no Processo de Regulação

- Solicitante: médico(a) que solicita vaga de TRS ambulatorial (HD ou DP).
- Regulador: médico(a) que atua na gestão do SUS, avalia a solicitação de diálise, verifica a pertinência técnica e a presença de documentos obrigatórios, e encaminha o caso para ser avaliado em uma unidade de diálise.
- Executante: médico(a) da unidade de diálise que avalia o paciente e seus exames e decide pela admissão ou não do paciente no serviço.

#### Tipos de Solicitação de TRS Ambulatorial

- Inicial: solicitação para paciente que não se encontra em programa de diálise crônica. Esse paciente pode estar ou não hospitalizado.
- Mudança de modalidade: solicitação de mudança entre as modalidades oferecidas no SUS: HD, DPA (diálise peritoneal automática), DPAC (diálise peritoneal ambulatorial contínua) e DPI (diálise peritoneal intermitente).
- Transferência: solicitação para paciente que deseja mudar seu tratamento para outra unidade de diálise.
- Trânsito: solicitação para realizar hemodiálise em outro município ou estado por período de até 30 dias.

## Resultados

Foram identificados 14 cenários com potencial divergência de opinião entre os profissionais envolvidos nos processos de regulação de acesso à TRS ambulatorial. A consulta pública ao documento inicial recebeu contribuições de 20 pessoas, dentre as quais: nove (45%) afirmaram concordar com as recomendações; nove (45%) concordaram parcialmente, apresentando sugestões; e duas (10%) discordaram, sem fornecer comentários específicos. Em uma das contribuições recebidas, sugeriu-se adicionar um cenário com potencial dúvida de regulação aos 14 inicialmente identificados. Após análise e revisão pelos autores, foram incorporadas 58% (14/24) das sugestões recebidas e elaboradas 15 recomendações.

### Regulação de Casos Com Suspeita ou Confirmação de Hepatites B e C

**Recomendação 1**. Pacientes com elevação de ALT/AST sem causa determinada e com HBsAg e/ou anti-HCV não reagentes necessitam de pesquisa de DNA-HBV e/ou RNA-HCV para admissão em unidade de diálise, independentemente do tipo de solicitação (inicial, mudança de modalidade, transferência ou trânsito). Da mesma forma, indivíduos com suspeita de hepatite B oculta (anti-HBc reagente, total ou IgM, com anti-HBs não reagente), mesmo sem elevação de transaminases, devem realizar testes de carga viral. Nesses casos, a obrigatoriedade de realizar os exames confirmatórios deve ser assumida pelo solicitante da vaga.

**Comentário**. Pacientes com DRC avançada apresentam habitualmente baixos níveis séricos de transaminases (alanina aminotransferase [ALT] e aspartato aminotransferase [AST])^15^. Nessa população, qualquer elevação de ALT ou AST acima dos valores de referência ou ≥ 50% em relação ao valor basal requer investigação^16^. As principais causas de elevação de transaminases são toxicidade por medicamentos, lesão muscular e infecções, entre outras^15^.

Dentre as infecções nos pacientes em HD, destacam-se as hepatites B e C, em razão do risco ocupacional e de transmissão cruzada nas unidades de diálise, do potencial de gravidade e da disponibilidade de tratamento específico^17,18^. A “janela imunológica” é comumente definida como o período entre a infecção por determinado agente biológico e sua detecção em exames de rotina. No caso da hepatite B, esse intervalo é de 30-60 dias até que o antígeno de superfície do vírus B (HBsAg) se torne reagente. Na hepatite C, o tempo entre a infecção e a detecção de anticorpos contra o vírus C (anti-HCV) varia entre 50-70 dias.

Nos casos em que o diagnóstico imediato é imperativo, como na admissão em unidades de diálise, indica-se a realização de exames capazes de detectar, em tempo real, uma infecção em curso, isto é, a carga viral (quantitativa ou qualitativa), também denominada reação em cadeia da polimerase (PCR), que identifica partículas do vírus B (DNA) ou do vírus C (RNA) no sangue ou em outros fluidos corporais^16^.

Para a regulação de acesso à TRS de indivíduos com anti-HCV reagente e/ou HBsAg reagente, recomendase, quando possível, que o solicitante realize os testes moleculares antes da admissão do paciente na unidade de diálise^17,18^. Também se recomenda que esses exames (carga viral por RNA-HCV e DNA-HBV) estejam disponíveis nessas unidades, com cobertura pelo SUS, tanto para as finalidades de diagnóstico quanto de acompanhamento.

Deve-se destacar que, no caso da hepatite C, o diagnóstico de doença ativa só pode ser estabelecido mediante a detecção da carga viral, ao contrário da hepatite B, em que a presença de HBsAg reagente é suficiente para confirmar infecção em curso^16,17^. Enquanto pacientes em diálise com RNA-HCV reagente apresentam alta taxa de cura uma vez instituído o tratamento adequado, aqueles com hepatite B confirmada podem requerer medicações com maior risco de eventos adversos e com menor taxa de resposta sustentada^17,18^.

### Paciente Internado em Condições de Alta Hospitalar, Assintomático e Com Cultura de Vigilância Positiva Para Bactérias Multirresistentes (BMR)

**Recomendação 2**. Não se deve aguardar a negativação de cultura de vigilância positiva para BMR em pacientes hospitalizados, assintomáticos e em condições de alta hospitalar para autorizar a admissão em unidades de diálise. Nesses estabelecimentos de saúde, os pacientes devem receber precaução de contato padrão, não havendo obrigatoriedade de realização do procedimento de HD em sala de isolamento.

**Comentário**. De acordo com recomendações da Agência Nacional de Vigilância Sanitária (ANVISA), todos os serviços de diálise devem possuir protocolos de prevenção e controle de infecções, bem como de monitorização e notificação de eventos adversos (incluindo infecções relacionadas à assistência à saúde)^19^. No caso do atendimento a pacientes colonizados/infectados por BMR, o uso de sala de isolamento, equipamentos de hemodiálise separados e trabalhadores exclusivos não é obrigatório. As medidas preventivas devem ser rigorosamente seguidas: higienização adequada das mãos, limpeza e desinfecção de superfícies, utilização de luvas e avental para todo contato com o paciente, e uso de óculos e máscara para proteção das mucosas dos olhos, boca e nariz dos profissionais de saúde, entre outras^19,20,21^.

### Paciente Internado em Hospital Privado, em Condições de Alta e Com Solicitação de Vaga de TRS Ambulatorial no SUS

**Recomendação 3**. Não se deve transferir o paciente para um hospital público com a finalidade exclusiva de formalizar o pedido de TRS ambulatorial, exceto por expressa vontade do paciente ou de seu responsável legal. Em hospitais privados, a solicitação de vaga de TRS ambulatorial no SUS deve ser encaminhada para a Secretaria Municipal de Saúde (SMS) do local de destino do paciente. Caberá à SMS informar ao hospital solicitante a lista de documentos, dados clínicos e exames obrigatórios, conforme as normas de regulação locais.

**Comentário**. Pacientes internados em hospitais privados podem ser dependentes exclusivos do SUS (como no caso de transplantados renais atendidos em hospitais privados que prestam serviços ao SUS, por exemplo), ter plano privado de saúde ou optar por custear a hospitalização com recursos próprios. No caso de pacientes internados que possuem plano privado de saúde, somente após esgotadas todas as possibilidades de contratualização da assistência em TRS entre o plano de saúde e uma unidade de diálise é que o hospital privado deverá enviar a solicitação de vaga de TRS no SUS para a Secretaria Municipal de Saúde (SMS). Independentemente desses cenários, o acesso ao SUS é um direito universal no Brasil, garantido a todo cidadão e em todo território nacional. A recomendação estabelece um fluxo de acesso à TRS ambulatorial em estabelecimentos de saúde privados, especialmente quando esses serviços não possuem acesso aos processos de regulação do SUS. A justificativa para recomendar que o ente municipal seja o receptor da solicitação de TRS ambulatorial está respaldada pelo princípio da descentralização do SUS e por portaria ministerial vigente^13,22^. É dever do hospital enviar à SMS todas as informações clínicas e documentos de acordo com as normas do SUS do município para o qual a vaga de TRS está sendo solicitada^13^. A SMS do município de destino do paciente deve receber a documentação completa e incluir o caso nos processos de regulação locais^13^. É imprescindível que haja uma comunicação clara e contínua entre a instituição hospitalar privada e o agente público regulador.

### Paciente em TRS Ambulatorial Financiada Por Plano Privado e Que Apresente Interrupção do Contrato Com a Saúde Suplementar

**Recomendação 4**. Não se recomenda encaminhar nenhum paciente em TRS para internação hospitalar exclusivamente em razão do cancelamento, voluntário ou involuntário, de seu plano privado de saúde. Após esgotadas as possibilidades de contratualização da assistência em TRS entre o plano privado e a unidade de diálise, esta deve enviar a solicitação de vaga de TRS no SUS para a Secretaria Municipal de Saúde (SMS) o mais brevemente possível.

**Comentário**. Existem diferentes formas de contratação de planos privados de saúde (individual, familiar ou empresarial) e diversos motivos para a interrupção desses contratos (inadimplência, mudança de plano, exclusão do beneficiário, demissão etc.). Em cada situação, o período em que a assistência à saúde deve ser mantida pelo plano de origem pode variar de acordo com as normas estabelecidas nos contratos e garantidas pela Agência Nacional de Saúde Suplementar. Em geral, esse prazo é de 30 a 60 dias^23,24,25^. Desse modo, para minimizar perdas de faturamento, recomenda-se que a unidade de diálise mantenha atualizadas as informações sobre os prazos de vigência dos contratos firmados entre os pacientes e os planos privados de saúde.

### Paciente Com Solicitação de Vaga de TRS Ambulatorial no SUS Proveniente de Outro Estado ou Outro País

**Recomendação 5**. O pedido de vaga de TRS no SUS deve ser encaminhado pelo serviço de saúde do estado ou país de origem do paciente diretamente para a SMS do local de destino solicitado. Caberá à SMS informar ao solicitante as normas de regulação locais, incluindo a lista completa de documentos, informações e exames obrigatórios.

**Comentário**. O princípio da universalidade do SUS assegura que o acesso aos serviços de saúde é um direito de todos os cidadãos brasileiros e de todas as pessoas que se encontram em território nacional, o que inclui brasileiros que se encontram em outros estados, estrangeiros, migrantes, imigrantes, refugiados e solicitantes de asilo^22,26^. A recomendação de encaminhar a solicitação eletiva de TRS ambulatorial diretamente à SMS de destino visa ordenar o fluxo, permitindo que o agente público inclua o caso na fila de regulação da mesma forma que faria com seus próprios munícipes^13,22^. Em situações de emergência, o Ministério da Saúde reconhece o direito à assistência à saúde mesmo para indivíduos que não possuem cadastro de pessoa física (CPF) ou registro geral (RG)^22^.

### Deve-Se Priorizar Alguns Tipos de Solicitações de TRS Ambulatorial em Detrimento a Outras?

**Recomendação 6**. Recomenda-se que seja seguida a seguinte ordem decrescente de prioridade: (1) solicitação inicial para paciente internado; (2) solicitação de mudança de modalidade; (3) solicitação inicial para paciente em domicílio; e (4) solicitação de transferência ou trânsito. Para pacientes em uma mesma situação entre as elencadas acima, sugere-se ordenar os critérios de prioridade do mais antigo para o mais novo.

**Comentário**. A ordenação do acesso a serviços de saúde utilizando critérios outros além da ordem cronológica da solicitação é uma prática do SUS e faz parte das ações do que comumente se denomina gestão ou qualificação de fila. Exemplos da aplicação de critérios clínicos de prioridade na fila para serviços de saúde são observados em assistências ambulatorial^27,28,29,30^, hospitalar^31^ e no contexto da TRS^32^. A consequência salutar desses processos de gestão é que haja diferentes tempos médios de espera de acordo com a condição atual do paciente e com o risco presumido de agravamento.

Pacientes que se encontram internados somente à espera de vaga de TRS ambulatorial possuem alto risco de desnutrição, infecção hospitalar, internação prolongada e óbito^33,34,35,36,37^. Ademais, a ocupação ociosa de um leito hospitalar prejudica o acesso de outros pacientes e eleva o custo operacional do sistema de saúde (prioridade 1).

As solicitações de mudança de modalidade de TRS ambulatorial, de hemodiálise para diálise peritoneal, frequentemente se dão em razão de falência de acesso vascular, infecções recorrentes de corrente sanguínea e instabilidade hemodinâmica^35^; a mudança de diálise peritoneal para hemodiálise pode ocorrer devido à peritonite ou à perda de eficiência de troca da membrana peritoneal^38,39,40^. Em ambos os casos, existe vulnerabilidade significativa e risco iminente de deterioração clínica (prioridade 2)^41,42^.

Reconhecemos que indivíduos com solicitação eletiva de TRS ambulatorial e que se encontram em domicílio apresentam risco de piora súbita da função renal e de internação por urgência dialítica, e que esse risco aumenta à medida que o tempo de espera pelo início de TRS se prolonga. Contudo, pela natureza da solicitação, o quadro clínico dessas pessoas é menos grave do que as condições descritas nas prioridades 1 e 2. Antes que a DRC dos indivíduos com solicitação eletiva de TRS se agrave, ainda é possível que essas pessoas procurem o serviço ambulatorial de origem para ajustes no plano terapêutico. Deste modo, tais pacientes devem manter seguimento nesse serviço para ajuste contínuo em sua terapia até a disponibilização de uma vaga de TRS (prioridade 3).

O atendimento tempestivo às solicitações de TRS ambulatorial por trânsito ou transferência é, sem dúvida, essencial por diversos motivos; apesar disso, a condição clínica dos requerentes é mais estável do que nas solicitações iniciais e de mudança de modalidade. Assim, sugere-se que as solicitações de trânsito ou transferência sejam classificadas como prioridade 4.

### O Executante Deve Repetir as Sorologias Virais Enviadas Pelo Solicitante (Hepatites B e C, e HIV) Antes da Admissão na Unidade de DiáLise?

**Recomendação 7**. Não se recomenda que o executante repita as sorologias virais (hepatites B e C, e HIV) enviadas pelo solicitante de forma rotineira ou sem justificativa válida.

**Comentário**. Os serviços de saúde de origem dos pacientes são os responsáveis diretos pela execução e validade dos exames realizados. Os órgãos públicos que fiscalizam as normas de funcionamento e a qualidade dos laboratórios de análises clínicas (públicos e privados) são os centros de vigilância sanitária estaduais e municipais^43^. A exigência de repetição de exames sorológicos válidos prejudica o fluxo regulatório de vagas, atrasa o início da TRS, pode ter impacto negativo na saúde do paciente e constitui uso inadequado de recursos, além de representar aumento de custos para o sistema de saúde. A lista de exames obrigatórios para admissão em unidade de diálise e os prazos recomendados de validade estão descritos na recomendação 8 deste consenso.

### Nas Solicitações de TRS Ambulatorial, Quais Exames Devem ser Obrigatoriamente Enviados Pelo Médico Solicitante ao Médico Regulador?

**Recomendação 8**. É de responsabilidade do solicitante enviar os resultados dos exames pactuados e respeitar um prazo máximo entre o resultado do exame e a data de avaliação pelo executante. Nas solicitações iniciais para pacientes internados, recomendase: hemograma, ureia, creatinina, sódio, potássio, glicemia, cálcio (total ou iônico) e ALT (validade de 15 dias); anti-HCV, anti-HBc-total, HBsAg, anti-HBs, anti-HIV e ultrassonografia renal (90 dias). Nas solicitações iniciais para pacientes em domicílio, recomenda-se: hemograma, ureia, creatinina, sódio, potássio, glicemia, cálcio (total ou iônico) e ALT (60 dias); anti-HCV, anti-HBc-total, HBsAg, anti-HBs, anti-HIV (180 dias) e ultrassonografia renal (360 dias). Nas solicitações de trânsito ou transferência, recomenda-se o envio dos exames mais recentes realizados na unidade de diálise de origem, com validade máxima equivalente à periodicidade prevista na diretriz brasileira vigente, isto é: hemoglobina, hematócrito, creatinina, sódio, potássio, cálcio (iônico ou total), fósforo, ALT, ureia (pré e pós) e glicemia (para pessoas com diabetes mellitus) - 30 dias; hemograma, saturação de transferrina, ferritina, fosfatase alcalina, paratormônio e hemoglobina glicada (se diabetes) - 90 dias; anti-HCV, anti-HBc-total, HBsAg, anti-HBs - 180 dias; e anti-HIV - 360 dias^44^.

**Comentário**. No caso das solicitações iniciais de TRS ambulatorial, os exames recomendados no processo de regulação devem garantir a possibilidade de o profissional executante realizar uma prescrição de diálise que seja segura para o paciente recém-admitido e para os demais pacientes da unidade de diálise de destino. Os exames obrigatórios devem, minimamente, permitir a confirmação do diagnóstico de DRC (ureia, creatinina, ultrassonografia renal)^12,13,44^, a prevenção de hipotensão arterial (hemoglobina/hematócrito)^45^, sangramentos (plaquetas)^46^ e distúrbios eletrolíticos (sódio, potássio, cálcio)^47^, além de possibilitar a redução de risco biológico ocupacional e de transmissão cruzada de hepatites e HIV (sorologias virais)16-18,48,49.

### Quais Documentos Devem SER Obrigatoriamente Enviados ao Médico Regulador Pelo Médico Solicitante de TRS Ambulatorial?

**Recomendação 9**. É dever do médico solicitante enviar relatório médico e os laudos contendo resultados dos exames obrigatórios, de acordo com a recomendação 8. O relatório médico deve ser completo, evolutivo e atualizado, contendo história clínica, medicações em uso, exame físico e, nos casos em que o paciente tenha iniciado TRS, o histórico de acessos para diálise (cateteres [venoso e peritoneal] e fístula arteriovenosa [FAV]), bem como a prescrição médica do procedimento (HD ou DP).

**Comentário**. É de responsabilidade do solicitante o envio completo das informações clínicas, exames laboratoriais e documentos obrigatórios pactuados no fluxo de regulação. O envio incompleto dessas informações retarda o aceite do paciente pela unidade executante, atrasa o início oportuno do tratamento dialítico e dificulta a prescrição segura do tratamento na nova unidade de diálise^45,46,47^.

### Quais Exames e Documentos Podem Ser Dispensados do Processo de Regulação de Acesso à TRS Ambulatorial?

**Recomendação 10**. Para fins de admissão em unidade de diálise, não se deve exigir do solicitante a apresentação dos resultados de exames como: fósforo, paratormônio, colesterol total e frações, triglicérides, ferro, ferritina, saturação de transferrina, eletrocardiograma, ecocardiograma, radiografia de tórax, sorologia para sífilis, entre outros. Da mesma forma, não deve ser condicionada a admissão à apresentação da carteira de vacinação do paciente nem a outros documentos e exames além dos indicados nas recomendações 8 e 9 deste documento.

**Comentário**. De acordo com as recomendações 8 e 9 deste consenso, deve-se exigir o mínimo de documentos e exames que permitam ao executante realizar a prescrição da diálise de maneira segura. Os exames e documentos exigidos no processo de regulação de acesso à TRS não têm por objetivo esgotar as demandas do paciente com DRC estágio 5, as quais deverão ser atendidas após a admissão na unidade de diálise^12,13,44^.

### Em Casos de Solicitação Inicial de HD, é Obrigação do Solicitante Realizar o Implante do Cateter Venoso Para HD?

**Recomendação 11**. Não se deve exigir do solicitante o implante de cateter venoso para HD em pacientes internados que estejam estáveis clinicamente, em condições de alta hospitalar, sem urgência dialítica e portadores de todos os exames e documentos obrigatórios. Nesse caso, o implante de cateter de HD não deve ser pré-requisito para alta hospitalar. Da mesma maneira, a unidade de diálise não pode recusar pacientes com DRC estágio 5 localizados em domicílio que estejam sem cateter venoso ou FAV funcionante.

**Comentário**. A referência para implante de cateteres venosos e confecção de FAV deve ser pactuada entre o gestor público e as unidades de diálise, como pré-requisito para o funcionamento desses estabelecimentos^13^. Esses procedimentos fazem parte da Tabela de Procedimentos, Medicamentos, Órteses, Próteses e Materiais do SUS (SIGTAP)^50^ e devem ser oferecidos pelo estabelecimento de saúde habilitado em HD, independentemente da localização do paciente (domicílio ou hospital)^13^.

O objetivo dessa recomendação é garantir o acesso oportuno de pessoas com DRC estágio 5 a uma unidade de diálise e não deve ser interpretada como contrária ao conceito inequívoco de que candidatos à HD devem, idealmente, confeccionar FAV antes da necessidade iminente de início de diálise^13,44^. Os hospitais com capacidade e experiência para a confecção de acessos vasculares podem colaborar com a rede local de serviços de saúde, sem que isso, porém, seja uma condição para o ingresso de pacientes nas unidades de diálise credenciadas ao SUS.

### Nos Casos de Solicitação Inicial de DP Para Pacientes Internados, Otreinamento e Implante de Cateter Peritoneal Devem Obrigatoriamente Acontecer Antes sa Alta Hospitalar?

**Recomendação 12**. Não se deve exigir do solicitante o implante de cateter peritoneal para pacientes internados em condições de alta hospitalar, que ainda não iniciaram TRS, sem urgência dialítica e portadores dos exames e documentos obrigatórios. Nesse caso, o implante de cateter peritoneal e o treinamento para DP não devem ser pré-requisitos para alta hospitalar.

**Comentário**. O treinamento para DP e o implante de cateter de *Tenckhoff* são procedimentos contidos na Tabela de Procedimentos do SUS e devem ser oferecidos pelo serviço de saúde habilitado em diálise peritoneal, independentemente da localização do paciente (domicílio ou hospital)^13,50^. Essa recomendação não se aplica a pacientes com qualquer condição clínica que exija hospitalização (instabilidade hemodinâmica, sepse grave, urgência dialítica, entre outras).

### O Serviço Executante Pode Recusar Pacientes Com Comorbidades que Impliquem Maior Complexidade Clínica?

**Recomendação 13**. Nenhum paciente que se encontre estável clinicamente, em condições de alta hospitalar ou localizado em domicílio, portador dos exames e documentos obrigatórios para regulação, pode ter sua admissão negada em razão exclusiva de idade avançada ou de condições de saúde que estejam dentro do perfil assistencial previamente pactuado entre os gestores e as unidades de diálise. Recomendase que os perfis assistenciais de cada unidade sejam claramente definidos, respeitando as limitações estruturais e garantindo a segurança do cuidado.

**Comentário**. O serviço de diálise integra a rede de assistência do SUS e não deve ser penalizado ao lhe ser atribuída a responsabilidade pelo tratamento integral do paciente em diálise. A porta de entrada do paciente com DRC, assim como de todo paciente do SUS, é a Atenção Primária à Saúde, a qual é responsável pelo encaminhamento para os serviços de média e alta complexidade e pela coordenação do cuidado integral^22^. Em caso de descompensação clínica durante o procedimento de hemodiálise, a unidade de diálise deve prestar o primeiro atendimento ao paciente, garantindo sua estabilização^13^. Cabe ao gestor público local assegurar acesso imediato à rede de urgência e emergência e a articulação entre os serviços de saúde para promover o cuidado compartilhado^13^.

### O Serviço Executante Pode Recusar Pacientes Privados de Liberdade?

**Recomendação 14**. A admissão de pacientes em unidade de diálise não pode ser negada ou postergada em razão de o paciente ser privado de liberdade.

**Comentário**. A Política Nacional de Atenção Integral à Saúde das Pessoas Privadas de Liberdade no Sistema Prisional, no âmbito do SUS (PNAISP), visa garantir acesso universal, equânime e contínuo às ações e serviços de saúde para pessoas privadas de liberdade^51^. Todos os estados e o Distrito Federal aderiram formalmente à PNAISP, devendo seguir suas diretrizes e os planos de ação estaduais e municipais para sua execução.

### Qual o Limite de Idade Que Define o Atendimento de Crianças em Diálise Ambulatorial?

**Recomendação 15**. A assistência em nefrologia pediátrica nas unidades de diálise deve seguir o limite de idade de até 12 anos completos.

**Comentário**. O Sistema de Gerenciamento da Tabela de Procedimentos, Medicamentos e OPM do SUS define a idade de 0 a 12 anos completos como aquela a quem se destinam os procedimentos relacionados à hemodiálise pediátrica^50^. Em acordo, a Portaria número 1675, de 2018, do Ministério da Saúde, que dispõe sobre os critérios para a organização, funcionamento e financiamento do cuidado à pessoa com DRC no SUS, especifica o atendimento em diálise pediátrica, o qual abrange a faixa etária de 0 a 12 anos^13^. Entretanto, deve-se reconhecer as necessidades específicas de pacientes com DRC avançada, com idades de 13 a 18 anos e baixo peso corporal. Essas pessoas podem requerer adaptações técnicas nas unidades de diálise e atendimento por nefrologista pediátrico. Recomendase que essa assistência seja pactuada entre o gestor público contratante e a unidade de diálise.

## Discussão

O presente consenso produziu 15 recomendações para apoiar a regulação de acesso à diálise ambulatorial no âmbito do SUS. Nas fases de elaboração e consulta pública, foi possível obter a participação de profissionais de saúde de todas as cinco regiões do Brasil.

A necessidade de padronizar as normas que regem o acesso à diálise ambulatorial é premente. Embora a maior parte das unidades de diálise no Brasil que prestam serviço ao SUS esteja sob gestão municipal^52^, observa-se intenso deslocamento intermunicipal e até interestadual de pessoas para tratamento em TRS^53^. Isso ocorre, entre outros fatores, em razão do alto percentual de municípios de pequeno porte^54^, das dimensões continentais do país e das iniquidades de oferta de serviços de saúde^55^.

As recomendações aqui apresentadas devem ser avaliadas respeitando as heterogeneidades regionais do Brasil. Em muitos locais, a escassez de unidades de diálise e a limitação de recursos físicos e humanos nos serviços solicitantes e órgãos reguladores certamente levarão a uma maior dificuldade de implementação do conteúdo integral deste documento^56^. Contudo, a título de exemplo, em que pese a indisponibilidade esperada de dosagens da carga viral de HBV e HCV, não encontramos alternativa segura para dispensar a realização desses exames no caso de pacientes com elevação de transaminases sem causa determinada e resultados negativos de HBsAg e anti-HCV, respectivamente.

Além disso, o uso de telerregulação deve ser incentivado como instrumento de suporte às equipes de regulação, especialmente em regiões com escassez de nefrologistas ou com dificuldades logísticas. Experiências nacionais e internacionais demonstram o potencial dessas ferramentas na ampliação do acesso e na qualificação dos processos gerenciais^57^.

Sugere-se que, para além da implementação das recomendações, os gestores públicos incorporem indicadores operacionais que permitam avaliar a eficiência dos processos regulatórios, como o tempo médio de espera (segundo tipo de solicitação, perfil sorológico, idade dos pacientes, entre outras variáveis), o percentual de solicitações devolvidas por documentação incompleta e, nos locais em que estiver disponível, a taxa de resolubilidade da telerregulação^55^. Além de eficiente, recomenda-se que o acesso à diálise ambulatorial seja transparente: o serviço de saúde solicitante da vaga de TRS deve ser informado se o pedido foi ou não recepcionado pela central de regulação de maneira completa; o paciente ou seu responsável legal podem requisitar informação sobre a posição na lista de espera por vaga de TRS; solicitantes e pacientes devem ter direito de conhecer o tempo médio de espera pelo atendimento em determinado município ou microrregião de saúde.

Globalmente, a forma como os indivíduos iniciam TRS reflete, em parte, a atenção à saúde nos níveis primário e secundário^1,2,4,9,11^. Entre outros, os seguintes indicadores devem ser de domínio público e das sociedades médicas: o percentual de início planejado de TRS, a taxa de início de HD com FAV funcionante e o percentual de utilização de DP como primeira modalidade de TRS. Essas e outras informações podem ser produzidas a partir de dados dos sistemas de regulação do SUS e são essenciais para monitorar a linha de cuidado às pessoas com DRC^58,59^.

Muitas das recomendações aqui apresentadas podem ser utilizadas nas contratualizações entre operadoras de saúde e unidades de diálise que atendem pessoas com planos privados. Como é de conhecimento geral, o Brasil possui um sistema de saúde duplo^60^, com intensa sobreposição de ações dos sistemas público e privado. Esta realidade pode ser comprovada pelas seguintes constatações: o órgão fiscalizador dos estabelecimentos de saúde, quer públicos quer privados, é a vigilância sanitária, uma instituição pertencente ao SUS^13,43^; o direito ao SUS é universal, independentemente de o indivíduo possuir ou não plano de saúde privado^22^; a assistência em oncologia e a TRS (diálise e transplante renal) são os serviços com maior utilização do SUS por beneficiários de planos privados de saúde^61,62^.

As normas que regem a obrigatoriedade da oferta de serviços pelos planos de saúde são definidas pela Agência Nacional de Saúde Suplementar (ANS) e pelas especificidades dos contratos celebrados com o beneficiário, como nível de assistência (ambulatorial ou hospitalar, eletiva ou de urgência), tipo de rede (própria ou credenciada), área geográfica de abrangência, entre outras^24,63^. Com o objetivo de responsabilizar legal e financeiramente os planos privados de saúde, a ANS, órgão vinculado ao Ministério da Saúde, emite ordens trimestrais de cobrança dos atendimentos de beneficiários ocorridos na rede do SUS^62^. Os valores não ressarcidos pelos planos privados são convertidos em dívida ativa junto ao cadastro federal de inadimplência e ficam sujeitos à cobrança judicial^63^.

Em conclusão, reconhece-se que o conteúdo integral deste documento não possui força de lei. Todavia, foi possível compilar opiniões de nefrologistas de todas as regiões do Brasil e sustentar a maior parte das recomendações por legislações brasileiras e literatura especializada. Portanto, este consenso poderá subsidiar pactuações entre gestores, solicitantes e executantes de TRS ambulatorial, tanto no SUS quanto na saúde suplementar. Recomendase que sejam conduzidos estudos futuros, preferencialmente em diferentes regiões do Brasil, para avaliar o impacto das recomendações propostas na eficiência do acesso à TRS ambulatorial.

## Data Availability

As sugestões recebidas na fase de consulta pública com as respectivas considerações dos autores estão disponíveis por meio de solicitação à Sociedade Brasileira de Nefrologia e mediante justificativa razoável (secretaria@sbn.org.br).
